# Ethyl 2,6-bis­(4-chloro­phen­yl)-4-(4-methyl­anilino)-1-(4-methyl­phen­yl)-1,2,5,6-tetra­hydro­pyridine-3-carboxyl­ate

**DOI:** 10.1107/S1600536813005126

**Published:** 2013-02-28

**Authors:** Sumati Anthal, Goutam Brahmachari, Suvankar Das, Rajni Kant, Vivek K. Gupta

**Affiliations:** a Post-Graduate Department of Physics & Electronics, University of Jammu, Jammu Tawi 180 006, India; b Laboratory of Natural Products & Organic Synthesis, Department of Chemistry, Visva-Bharati University, Santiniketan 731 235, West Bengal, India

## Abstract

In the title mol­ecule, C_34_H_32_Cl_2_N_2_O_2_, the tetra­hydro­pyridine ring adopts a distorted boat conformation and both 4-chloro­phenyl substituents are in axial positions. An intra­molecular N—H⋯O hydrogen bond is formed by the amino group and carbonyl O atom. In the crystal, weak C—H⋯Cl inter­actions link the mol­ecules into chains along [010].

## Related literature

For general background to functionalized piperidines, see: Pinder (1992 ▶); Watson *et al.* (2000 ▶); Breman *et al.* (2001 ▶); Kamei *et al.* (2005 ▶). For related structures, see: Sambyal *et al.* (2011 ▶); Brahmachari & Das (2012 ▶); Khan *et al.* (2010 ▶); Anthal *et al.* (2013 ▶). For ring conformations, see: Duax & Norton (1975 ▶).
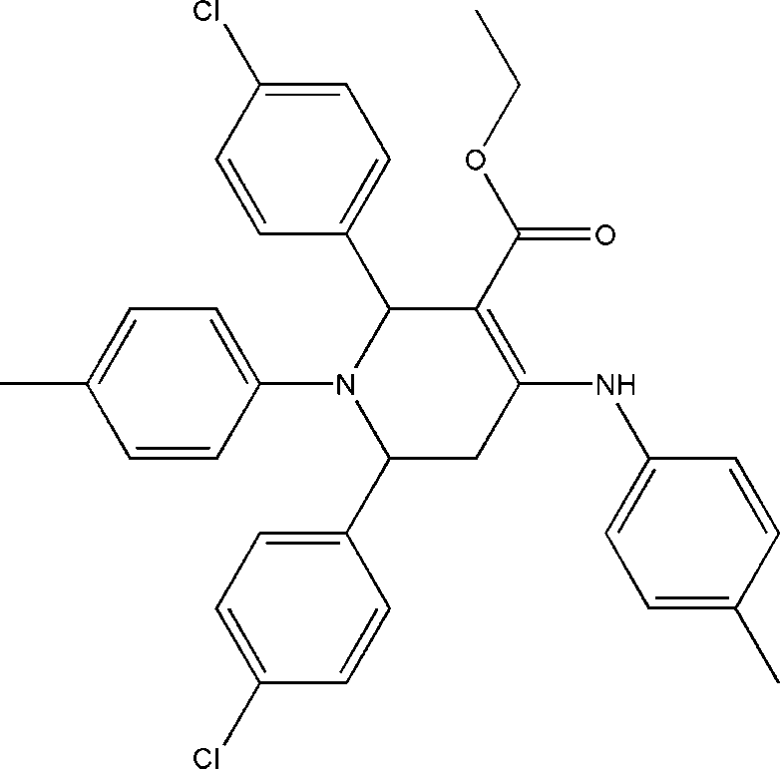


## Experimental

### 

#### Crystal data

C_34_H_32_Cl_2_N_2_O_2_
*M* *_r_* = 571.52
Triclinic, 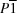

*a* = 10.0851 (6) Å
*b* = 12.4340 (8) Å
*c* = 14.2414 (10) Å
α = 113.405 (6)°
β = 102.233 (5)°
γ = 101.194 (5)°
*V* = 1522.63 (17) Å^3^
*Z* = 2
Mo *K*α radiation
μ = 0.25 mm^−1^
*T* = 293 K
0.30 × 0.20 × 0.20 mm

#### Data collection

Oxford Diffraction Xcalibur Sapphire3 diffractometer
Absorption correction: multi-scan (*CrysAlis PRO*; Oxford Diffraction, 2010 ▶) *T*_min_ = 0.853, *T*_max_ = 1.000
13336 measured reflections
5342 independent reflections
2181 reflections with *I* > 2σ(*I*)
*R*_int_ = 0.059

#### Refinement

*R*[*F* ^2^ > 2σ(*F* ^2^)] = 0.053
*wR*(*F* ^2^) = 0.109
*S* = 0.89
5342 reflections
365 parameters
H-atom parameters constrained
Δρ_max_ = 0.25 e Å^−3^
Δρ_min_ = −0.21 e Å^−3^

### 

Data collection: *CrysAlis PRO* (Oxford Diffraction, 2010 ▶); cell refinement: *CrysAlis PRO*; data reduction: *CrysAlis PRO*; program(s) used to solve structure: *SHELXS97* (Sheldrick, 2008 ▶); program(s) used to refine structure: *SHELXL97* (Sheldrick, 2008 ▶); molecular graphics: *ORTEP-3 for Windows* (Farrugia, 2012 ▶); software used to prepare material for publication: *PLATON* (Spek, 2009 ▶).

## Supplementary Material

Crystal structure: contains datablock(s) I, global. DOI: 10.1107/S1600536813005126/gk2555sup1.cif

Structure factors: contains datablock(s) I. DOI: 10.1107/S1600536813005126/gk2555Isup2.hkl

Supplementary material file. DOI: 10.1107/S1600536813005126/gk2555Isup3.cml

Additional supplementary materials:  crystallographic information; 3D view; checkCIF report

## Figures and Tables

**Table 1:** Hydrogen-bond geometry (Å, °)

*D*—H⋯*A*	*D*—H	H⋯*A*	*D*⋯*A*	*D*—H⋯*A*
N2—H2⋯O1	0.86	2.10	2.707 (4)	127
C9—H9*A*⋯Cl2^i^	0.96	2.74	3.698 (4)	173

Symmetry code: (i) 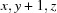.

**Fig. 1.**
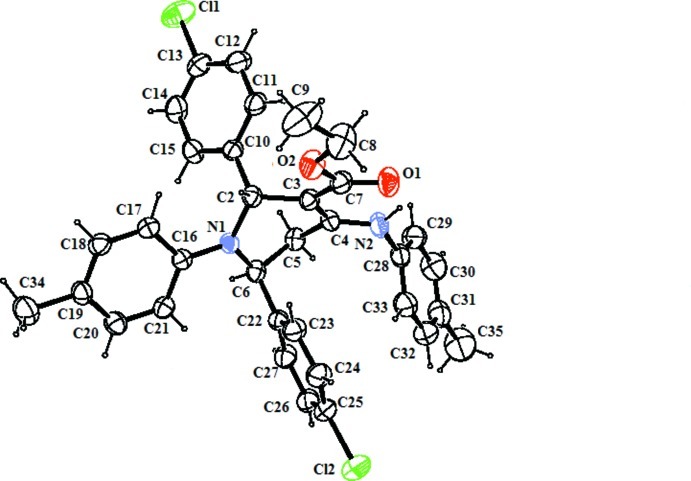
ORTEP view of the title molecule with the atom-labeling scheme. The displacement ellipsoids are drawn at the 40% probability level. H atoms are shown as small spheres of arbitrary radii.

**Fig. 2.**
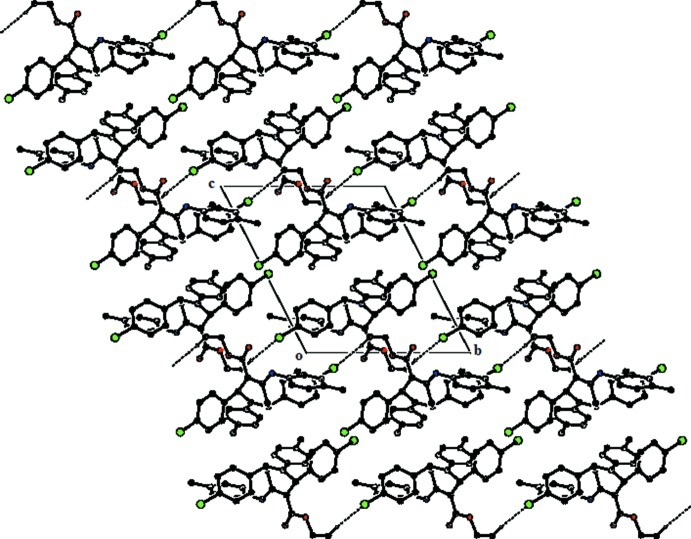
The packing arrangement of molecules viewed down the a axis.

## supplementary crystallographic information

### Comment

Functionalized piperidine scaffolds are the attractive structural motifs as they are found to constitute a pharmaceutically significant skeleton in numerous natural products (Pinder, 1992), synthetic pharmaceuticals (Breman *et al.*, 2001; Watson *et al.*, 2000), and a wide variety of biologically active compounds. A large number of compounds bearing piperidine scaffold have entered into preclinical and clinical trials over the last few years (Kamei *et al.*, 2005). Continuing our interest in densely functionalized piperidines (Sambyal *et al.*, 2011; Brahmachari & Das, 2012) we present here the crystal structure of the title compound (Fig. 1). Molecular structure of the title compound is similar to that of closely related structures (Khan *et al.*, 2010; Anthal *et al.*, 2013). The tetrahydropyridine ring adopts distorted boat conformation with asymmetry parameters [ΔC_s_(C2)=11.1] and [ΔC_s_(C3—C4)=16.9] (Duax *et al.*, 1975). The dihedral angles between two 4-chlorophenyl rings[(C10-C15) and (C22-C27)] and two 4-tolyl rings[(C16-C21) and (C28-C33)]are 47.7 (1)° and 48.5 (1)° respectively. The dihedral angle between 4-chlorophenyl ring (C10-C15) and 4-tolyl ring (C28-C33) is 80.4 (1)°, between 4-tolyl ring (C16-C21) and 4-chlorophenyl ring (C22-C27) is 88.7 (1)°, between 4-chlorophenyl ring (C22-C27) and 4-tolyl ring (C28-C33) is 41.4 (1)° and 4-chlorophenyl ring (C10-C15) makes a dihedral angle of 68.7 (1)° with 4-tolyl ring (C16-C21). In the crystal, an intramolecular hydrogen bond N2—H2···O1 is found. The amino N1 atom links through H1 to the oxygen of the carbonyl group. This interaction leads to the formation of a pseudo-six membered ring comprising atoms O1, C7, C3, C4, N2 and H2. Packing view of the molecules in the unit cell viewed down the *a* axis is shown in Fig.2. Weak intermolecular C—H···Cl interactions (Table 1) link the molecules into chains parallel to [010].

### Experimental

An oven-dried screw cap reaction tube was charged with a magnetic stir bar, 4-methylaniline (2 mmol), ethyl acetoacetate (1 mmol) and Bi(NO_3_)_3_.5H_2_O (10 mol%) in 4 ml ethanol; the mixture was stirred at room temperature for 20 min, and after then 4-chlorobenzaldehyde (2 mmol) was added to the reaction mixture and stirring was continued up to 21 h to complete the reaction (monitored by TLC). On completion of the reaction, a thick white precipitate was obtained. The solid residue was filtered off and washed with cold ethanol-water. The solid mass was dissolved in hot ethyl acetate-ethanol mixture and filtered off when bismuth salt separated out; the filtrate on standing afforded white crystals of the title compound, characterized by elemental analyses and spectral studies including FT—IR, ^1^H-NMR, and ^13^C-NMR. For X-ray study single crystals were prepared by further recrystallization by slow evaporation from ethanol-ethyl acetate-water solution (colourless crystals; mp 500–502 K; anal. calcd for C_34_H_32_Cl_2_N_2_O_2_: C 71.45, H 5.64, N 4.90; found: C 71.42, H 5.66, N 4.91).

### Refinement

All H atoms were positioned geometrically and were treated as riding on their parent C/N atoms, with C—H distances of 0.93–0.98 Å and N—H distance of 0.86 Å; *U*_iso_(H) = 1.2*U*_eq_(C/N), except for the methyl groups where *U*_iso_(H) = 1.5*U*_eq_(C).

### Crystal data

**Table:**

C_34_H_32_Cl_2_N_2_O_2_	*Z* = 2
*M**_r_* = 571.52	*F*(000) = 600
Triclinic, *P*1	*D*_x_ = 1.247 Mg m^−^^3^
Hall symbol: -P 1	Mo *K*α radiation, λ = 0.71073 Å
*a* = 10.0851 (6) Å	Cell parameters from 3234 reflections
*b* = 12.4340 (8) Å	θ = 3.3–28.9°
*c* = 14.2414 (10) Å	µ = 0.25 mm^−^^1^
α = 113.405 (6)°	*T* = 293 K
β = 102.233 (5)°	Block-shaped, white
γ = 101.194 (5)°	0.30 × 0.20 × 0.20 mm
*V* = 1522.63 (17) Å^3^	

### Data collection

**Table:**

Oxford Diffraction Xcalibur Sapphire3 diffractometer	5342 independent reflections
Radiation source: fine-focus sealed tube	2181 reflections with *I* > 2σ(*I*)
Graphite monochromator	*R*_int_ = 0.059
Detector resolution: 16.1049 pixels mm^-1^	θ_max_ = 25.0°, θ_min_ = 3.3°
ω scans	*h* = −11→11
Absorption correction: multi-scan (*CrysAlis PRO*; Oxford Diffraction, 2010)	*k* = −14→14
*T*_min_ = 0.853, *T*_max_ = 1.000	*l* = −16→16
13336 measured reflections	

### Refinement

**Table:**

Refinement on *F*^2^	Secondary atom site location: difference Fourier map
Least-squares matrix: full	Hydrogen site location: inferred from neighbouring sites
*R*[*F*^2^ > 2σ(*F*^2^)] = 0.053	H-atom parameters constrained
*wR*(*F*^2^) = 0.109	*w* = 1/[σ^2^(*F*_o_^2^) + (0.0203*P*)^2^] where *P* = (*F*_o_^2^ + 2*F*_c_^2^)/3
*S* = 0.89	(Δ/σ)_max_ = 0.001
5342 reflections	Δρ_max_ = 0.25 e Å^−^^3^
365 parameters	Δρ_min_ = −0.21 e Å^−^^3^
0 restraints	Extinction correction: *SHELXL97* (Sheldrick, 2008), Fc^*^=kFc[1+0.001xFc^2^λ^3^/sin(2θ)]^-1/4^
Primary atom site location: structure-invariant direct methods	Extinction coefficient: 0.0060 (5)

### Special details

**Table:**

Experimental. ^1^H-NMR (400 MHz, CDCl_3_): δ_H_ 1.46 (t, *J* = 6.8 Hz, 3H), 2.16 (s, 3H), 2.27 (s, 3H), 2.70 (dd, *J* = 2.4, 15.2 Hz, 1H), 2.77 (dd, *J* = 5.2, 15.2 Hz, 1H), 4.29–4.33 (m, 1H), 4.35–4.44 (m, 1H), 5.05 (br s, 1H), 6.29 (t, *J* = 8, 11.2 Hz, 3H), 6.36 (d, *J* = 8.8 Hz, 2H), 6.88 (d, *J* = 8.4 Hz, 2H), 6.94 (d, *J* = 8 Hz, 2H), 7.05(d, *J* = 8.4 Hz, 2H), 7.21–7.26 (m, 6H), 10.20 (br s, 1H). ^13^C-NMR (100 MHz, CDCl_3_): δ_C_ 14.82, 20.13, 20.92, 33.62, 54.84, 57.35, 59.74, 97.24, 113.0, 125.77, 125.83, 127.85, 128.07, 128.35, 128.73, 129.59, 132.01, 132.76, 135.01, 135.88, 141.23, 142.80, 144.37, 156.16, 168.03. IR ν_max_ (KBr): 3242, 3024, 2974, 2915, 2870, 1653, 1589, 1514, 1483, 1256, 1172, 1076, 1012, 804, 678 cm^-1^.*CrysAlis PRO*, Oxford Diffraction Ltd., Version 1.171.34.40 (release 27–08-2010 CrysAlis171. NET) (compiled Aug 27 2010,11:50:40) Empirical absorption correction using spherical harmonics, implemented in SCALE3 ABSPACK scaling algorithm.
Geometry. All e.s.d.'s (except the e.s.d. in the dihedral angle between two l.s. planes) are estimated using the full covariance matrix. The cell e.s.d.'s are taken into account individually in the estimation of e.s.d.'s in distances, angles and torsion angles; correlations between e.s.d.'s in cell parameters are only used when they are defined by crystal symmetry. An approximate (isotropic) treatment of cell e.s.d.'s is used for estimating e.s.d.'s involving l.s. planes.
Refinement. Refinement of *F*^2^ against ALL reflections. The weighted *R*-factor *wR* and goodness of fit *S* are based on *F*^2^, conventional *R*-factors *R* are based on *F*, with *F* set to zero for negative *F*^2^. The threshold expression of *F*^2^ > σ(*F*^2^) is used only for calculating *R*-factors(gt) *etc*. and is not relevant to the choice of reflections for refinement. *R*-factors based on *F*^2^ are statistically about twice as large as those based on *F*, and *R*- factors based on ALL data will be even larger.

### Fractional atomic coordinates and isotropic or equivalent isotropic displacement parameters (Å^2^)

**Table:**

	*x*	*y*	*z*	*U*_iso_*/*U*_eq_	
Cl1	0.22422 (13)	1.01537 (10)	0.47443 (10)	0.1168 (5)	
Cl2	0.36062 (12)	−0.11737 (10)	0.09237 (10)	0.1157 (5)	
O1	0.0417 (3)	0.3327 (2)	−0.03361 (19)	0.0828 (8)	
O2	0.2464 (2)	0.4814 (2)	0.01502 (18)	0.0734 (7)	
N1	0.4134 (2)	0.4914 (2)	0.29596 (19)	0.0496 (7)	
N2	−0.0058 (3)	0.2735 (2)	0.1225 (2)	0.0689 (9)	
H2	−0.0501	0.2706	0.0625	0.083*	
C2	0.3304 (3)	0.5348 (3)	0.2295 (2)	0.0493 (8)	
H2A	0.3889	0.5539	0.1880	0.059*	
C3	0.1954 (3)	0.4340 (3)	0.1478 (3)	0.0488 (8)	
C4	0.1191 (3)	0.3651 (3)	0.1837 (3)	0.0532 (9)	
C5	0.1815 (3)	0.3929 (3)	0.2988 (2)	0.0564 (9)	
H5A	0.1303	0.3288	0.3122	0.068*	
H5B	0.1728	0.4708	0.3463	0.068*	
C6	0.3382 (3)	0.3997 (3)	0.3215 (3)	0.0510 (9)	
H6	0.3792	0.4284	0.3993	0.061*	
C7	0.1516 (4)	0.4089 (3)	0.0359 (3)	0.0586 (10)	
C8	0.2095 (4)	0.4698 (4)	−0.0935 (3)	0.0985 (14)	
H8A	0.2042	0.3881	−0.1448	0.118*	
H8B	0.1170	0.4815	−0.1128	0.118*	
C9	0.3167 (5)	0.5614 (3)	−0.0972 (3)	0.1185 (16)	
H9A	0.3235	0.6418	−0.0446	0.178*	
H9B	0.2912	0.5567	−0.1679	0.178*	
H9C	0.4070	0.5469	−0.0815	0.178*	
C10	0.3019 (3)	0.6540 (3)	0.2953 (3)	0.0507 (9)	
C11	0.2103 (3)	0.6973 (3)	0.2428 (3)	0.0648 (10)	
H11	0.1642	0.6512	0.1688	0.078*	
C12	0.1861 (4)	0.8064 (4)	0.2976 (3)	0.0746 (11)	
H12	0.1236	0.8330	0.2605	0.090*	
C13	0.2527 (4)	0.8763 (3)	0.4059 (3)	0.0688 (11)	
C14	0.3449 (4)	0.8373 (3)	0.4613 (3)	0.0729 (11)	
H14	0.3911	0.8849	0.5352	0.087*	
C15	0.3683 (3)	0.7264 (3)	0.4058 (3)	0.0608 (10)	
H15	0.4300	0.6998	0.4435	0.073*	
C16	0.5610 (3)	0.5394 (3)	0.3382 (2)	0.0447 (8)	
C17	0.6367 (3)	0.6453 (3)	0.3357 (2)	0.0524 (9)	
H17	0.5870	0.6842	0.3038	0.063*	
C18	0.7817 (3)	0.6921 (3)	0.3795 (3)	0.0573 (9)	
H18	0.8281	0.7620	0.3758	0.069*	
C19	0.8621 (3)	0.6397 (3)	0.4289 (3)	0.0580 (9)	
C20	0.7892 (3)	0.5358 (3)	0.4315 (3)	0.0606 (10)	
H20	0.8403	0.4981	0.4641	0.073*	
C21	0.6432 (3)	0.4858 (3)	0.3877 (2)	0.0528 (9)	
H21	0.5981	0.4152	0.3909	0.063*	
C22	0.3522 (3)	0.2723 (3)	0.2641 (3)	0.0495 (9)	
C23	0.3729 (3)	0.2285 (3)	0.1648 (3)	0.0624 (10)	
H23	0.3852	0.2806	0.1331	0.075*	
C24	0.3758 (3)	0.1092 (3)	0.1110 (3)	0.0707 (11)	
H24	0.3888	0.0809	0.0437	0.085*	
C25	0.3591 (4)	0.0333 (3)	0.1587 (4)	0.0746 (12)	
C26	0.3418 (3)	0.0744 (4)	0.2592 (3)	0.0734 (12)	
H26	0.3337	0.0229	0.2918	0.088*	
C27	0.3366 (3)	0.1935 (3)	0.3110 (3)	0.0645 (10)	
H27	0.3225	0.2211	0.3780	0.077*	
C28	−0.0709 (3)	0.1806 (3)	0.1490 (3)	0.0627 (10)	
C29	−0.1797 (4)	0.1888 (3)	0.1914 (3)	0.0768 (12)	
H29	−0.2129	0.2565	0.2048	0.092*	
C30	−0.2411 (4)	0.0965 (4)	0.2146 (3)	0.0907 (14)	
H30	−0.3161	0.1024	0.2427	0.109*	
C31	−0.1929 (5)	−0.0037 (4)	0.1966 (4)	0.0874 (14)	
C32	−0.0836 (4)	−0.0106 (4)	0.1543 (3)	0.0900 (14)	
H32	−0.0503	−0.0782	0.1407	0.108*	
C33	−0.0212 (4)	0.0819 (4)	0.1314 (3)	0.0789 (12)	
H33	0.0545	0.0767	0.1040	0.095*	
C34	1.0228 (3)	0.6960 (3)	0.4809 (3)	0.0972 (14)	
H34A	1.0533	0.6767	0.5393	0.146*	
H34B	1.0482	0.7839	0.5082	0.146*	
H34C	1.0686	0.6630	0.4282	0.146*	
C35	−0.2600 (4)	−0.1064 (4)	0.2214 (4)	0.145 (2)	
H35A	−0.3102	−0.1804	0.1551	0.217*	
H35B	−0.3255	−0.0821	0.2596	0.217*	
H35C	−0.1865	−0.1212	0.2653	0.217*	

### Atomic displacement parameters (Å^2^)

**Table:**

	*U*^11^	*U*^22^	*U*^33^	*U*^12^	*U*^13^	*U*^23^
Cl1	0.1645 (12)	0.0713 (8)	0.1186 (11)	0.0572 (8)	0.0661 (9)	0.0273 (8)
Cl2	0.1328 (10)	0.0596 (8)	0.1190 (11)	0.0344 (7)	0.0103 (8)	0.0197 (7)
O1	0.0872 (19)	0.0779 (19)	0.0520 (17)	−0.0019 (15)	−0.0075 (14)	0.0274 (16)
O2	0.0933 (18)	0.0777 (18)	0.0403 (15)	0.0102 (14)	0.0167 (13)	0.0285 (14)
N1	0.0462 (16)	0.0515 (18)	0.0501 (18)	0.0096 (13)	0.0088 (13)	0.0281 (16)
N2	0.0542 (18)	0.072 (2)	0.065 (2)	0.0005 (16)	−0.0024 (15)	0.0360 (19)
C2	0.052 (2)	0.052 (2)	0.039 (2)	0.0114 (17)	0.0099 (16)	0.0212 (19)
C3	0.055 (2)	0.047 (2)	0.039 (2)	0.0123 (17)	0.0119 (17)	0.0171 (18)
C4	0.046 (2)	0.056 (2)	0.049 (2)	0.0140 (18)	0.0053 (18)	0.021 (2)
C5	0.055 (2)	0.058 (2)	0.050 (2)	0.0096 (17)	0.0156 (18)	0.022 (2)
C6	0.054 (2)	0.053 (2)	0.039 (2)	0.0085 (17)	0.0109 (16)	0.0204 (19)
C7	0.072 (3)	0.052 (2)	0.046 (2)	0.016 (2)	0.013 (2)	0.020 (2)
C8	0.129 (4)	0.103 (4)	0.050 (3)	0.007 (3)	0.026 (2)	0.037 (3)
C9	0.209 (5)	0.080 (3)	0.077 (3)	0.037 (3)	0.069 (3)	0.038 (3)
C10	0.055 (2)	0.047 (2)	0.047 (2)	0.0133 (17)	0.0141 (17)	0.022 (2)
C11	0.078 (3)	0.052 (2)	0.052 (3)	0.0124 (19)	0.0099 (19)	0.021 (2)
C12	0.093 (3)	0.059 (3)	0.076 (3)	0.029 (2)	0.025 (3)	0.034 (3)
C13	0.080 (3)	0.049 (3)	0.072 (3)	0.020 (2)	0.030 (2)	0.019 (2)
C14	0.069 (3)	0.074 (3)	0.053 (3)	0.018 (2)	0.020 (2)	0.009 (2)
C15	0.057 (2)	0.071 (3)	0.046 (2)	0.0229 (19)	0.0156 (18)	0.017 (2)
C16	0.052 (2)	0.047 (2)	0.034 (2)	0.0149 (17)	0.0142 (16)	0.0169 (18)
C17	0.057 (2)	0.047 (2)	0.053 (2)	0.0179 (17)	0.0193 (18)	0.0213 (19)
C18	0.057 (2)	0.052 (2)	0.069 (3)	0.0158 (18)	0.0256 (19)	0.030 (2)
C19	0.046 (2)	0.062 (3)	0.062 (3)	0.0144 (18)	0.0170 (18)	0.025 (2)
C20	0.059 (2)	0.069 (3)	0.056 (2)	0.0197 (19)	0.0160 (19)	0.031 (2)
C21	0.059 (2)	0.050 (2)	0.049 (2)	0.0118 (17)	0.0175 (17)	0.0234 (19)
C22	0.052 (2)	0.047 (2)	0.047 (2)	0.0128 (16)	0.0126 (17)	0.022 (2)
C23	0.073 (2)	0.059 (3)	0.054 (3)	0.022 (2)	0.0195 (19)	0.024 (2)
C24	0.085 (3)	0.067 (3)	0.054 (3)	0.031 (2)	0.022 (2)	0.019 (2)
C25	0.080 (3)	0.047 (3)	0.076 (3)	0.017 (2)	0.003 (2)	0.021 (3)
C26	0.077 (3)	0.058 (3)	0.076 (3)	0.008 (2)	0.003 (2)	0.038 (3)
C27	0.071 (2)	0.060 (3)	0.060 (3)	0.011 (2)	0.0174 (19)	0.032 (2)
C28	0.050 (2)	0.065 (3)	0.061 (3)	0.005 (2)	0.0048 (19)	0.030 (2)
C29	0.072 (3)	0.076 (3)	0.088 (3)	0.023 (2)	0.028 (2)	0.041 (3)
C30	0.079 (3)	0.109 (4)	0.095 (4)	0.020 (3)	0.034 (2)	0.057 (3)
C31	0.075 (3)	0.094 (4)	0.086 (4)	0.003 (3)	0.006 (3)	0.055 (3)
C32	0.092 (3)	0.068 (3)	0.099 (4)	0.018 (3)	0.010 (3)	0.042 (3)
C33	0.065 (3)	0.082 (3)	0.088 (3)	0.020 (2)	0.021 (2)	0.040 (3)
C34	0.058 (3)	0.107 (4)	0.132 (4)	0.021 (2)	0.023 (2)	0.066 (3)
C35	0.143 (4)	0.142 (5)	0.169 (5)	0.002 (3)	0.030 (4)	0.118 (4)

### Geometric parameters (Å, º)

**Table:**

Cl1—C13	1.731 (4)	C16—C21	1.403 (3)
Cl2—C25	1.737 (4)	C16—C17	1.405 (3)
O1—C7	1.220 (3)	C17—C18	1.364 (3)
O2—C7	1.347 (3)	C17—H17	0.9300
O2—C8	1.452 (3)	C18—C19	1.381 (4)
N1—C16	1.388 (3)	C18—H18	0.9300
N1—C6	1.455 (3)	C19—C20	1.376 (4)
N1—C2	1.460 (3)	C19—C34	1.518 (4)
N2—C4	1.354 (3)	C20—C21	1.375 (4)
N2—C28	1.437 (4)	C20—H20	0.9300
N2—H2	0.8600	C21—H21	0.9300
C2—C3	1.512 (4)	C22—C23	1.378 (4)
C2—C10	1.528 (4)	C22—C27	1.391 (4)
C2—H2A	0.9800	C23—C24	1.383 (4)
C3—C4	1.359 (4)	C23—H23	0.9300
C3—C7	1.443 (4)	C24—C25	1.369 (4)
C4—C5	1.494 (4)	C24—H24	0.9300
C5—C6	1.522 (3)	C25—C26	1.378 (5)
C5—H5A	0.9700	C26—C27	1.384 (4)
C5—H5B	0.9700	C26—H26	0.9300
C6—C22	1.519 (4)	C27—H27	0.9300
C6—H6	0.9800	C28—C29	1.362 (4)
C8—C9	1.436 (4)	C28—C33	1.367 (4)
C8—H8A	0.9700	C29—C30	1.387 (4)
C8—H8B	0.9700	C29—H29	0.9300
C9—H9A	0.9600	C30—C31	1.374 (5)
C9—H9B	0.9600	C30—H30	0.9300
C9—H9C	0.9600	C31—C32	1.365 (5)
C10—C11	1.386 (4)	C31—C35	1.529 (4)
C10—C15	1.389 (4)	C32—C33	1.389 (4)
C11—C12	1.370 (4)	C32—H32	0.9300
C11—H11	0.9300	C33—H33	0.9300
C12—C13	1.361 (4)	C34—H34A	0.9600
C12—H12	0.9300	C34—H34B	0.9600
C13—C14	1.374 (4)	C34—H34C	0.9600
C14—C15	1.386 (4)	C35—H35A	0.9600
C14—H14	0.9300	C35—H35B	0.9600
C15—H15	0.9300	C35—H35C	0.9600
			
C7—O2—C8	117.1 (3)	N1—C16—C17	122.3 (3)
C16—N1—C6	119.9 (2)	C21—C16—C17	116.1 (3)
C16—N1—C2	121.2 (2)	C18—C17—C16	121.2 (3)
C6—N1—C2	118.8 (2)	C18—C17—H17	119.4
C4—N2—C28	125.8 (3)	C16—C17—H17	119.4
C4—N2—H2	117.1	C17—C18—C19	122.5 (3)
C28—N2—H2	117.1	C17—C18—H18	118.8
N1—C2—C3	111.4 (2)	C19—C18—H18	118.8
N1—C2—C10	113.5 (2)	C20—C19—C18	116.8 (3)
C3—C2—C10	112.5 (2)	C20—C19—C34	121.4 (3)
N1—C2—H2A	106.3	C18—C19—C34	121.7 (3)
C3—C2—H2A	106.3	C21—C20—C19	122.2 (3)
C10—C2—H2A	106.3	C21—C20—H20	118.9
C4—C3—C7	121.3 (3)	C19—C20—H20	118.9
C4—C3—C2	116.8 (3)	C20—C21—C16	121.2 (3)
C7—C3—C2	121.9 (3)	C20—C21—H21	119.4
N2—C4—C3	124.8 (3)	C16—C21—H21	119.4
N2—C4—C5	118.9 (3)	C23—C22—C27	118.2 (3)
C3—C4—C5	116.4 (3)	C23—C22—C6	123.0 (3)
C4—C5—C6	109.2 (3)	C27—C22—C6	118.8 (3)
C4—C5—H5A	109.8	C22—C23—C24	121.7 (3)
C6—C5—H5A	109.8	C22—C23—H23	119.1
C4—C5—H5B	109.8	C24—C23—H23	119.1
C6—C5—H5B	109.8	C25—C24—C23	118.9 (4)
H5A—C5—H5B	108.3	C25—C24—H24	120.6
N1—C6—C22	114.5 (3)	C23—C24—H24	120.6
N1—C6—C5	109.8 (2)	C24—C25—C26	121.2 (4)
C22—C6—C5	110.3 (2)	C24—C25—Cl2	119.8 (4)
N1—C6—H6	107.3	C26—C25—Cl2	119.0 (3)
C22—C6—H6	107.3	C25—C26—C27	119.1 (3)
C5—C6—H6	107.3	C25—C26—H26	120.4
O1—C7—O2	122.2 (3)	C27—C26—H26	120.4
O1—C7—C3	125.6 (3)	C26—C27—C22	120.8 (4)
O2—C7—C3	112.2 (3)	C26—C27—H27	119.6
C9—C8—O2	108.8 (3)	C22—C27—H27	119.6
C9—C8—H8A	109.9	C29—C28—C33	119.5 (3)
O2—C8—H8A	109.9	C29—C28—N2	121.8 (3)
C9—C8—H8B	109.9	C33—C28—N2	118.7 (3)
O2—C8—H8B	109.9	C28—C29—C30	120.1 (4)
H8A—C8—H8B	108.3	C28—C29—H29	120.0
C8—C9—H9A	109.5	C30—C29—H29	120.0
C8—C9—H9B	109.5	C31—C30—C29	121.1 (4)
H9A—C9—H9B	109.5	C31—C30—H30	119.5
C8—C9—H9C	109.5	C29—C30—H30	119.5
H9A—C9—H9C	109.5	C32—C31—C30	118.2 (4)
H9B—C9—H9C	109.5	C32—C31—C35	120.2 (4)
C11—C10—C15	117.0 (3)	C30—C31—C35	121.6 (4)
C11—C10—C2	119.3 (3)	C31—C32—C33	121.0 (4)
C15—C10—C2	123.6 (3)	C31—C32—H32	119.5
C12—C11—C10	121.5 (3)	C33—C32—H32	119.5
C12—C11—H11	119.3	C28—C33—C32	120.1 (4)
C10—C11—H11	119.3	C28—C33—H33	119.9
C13—C12—C11	120.7 (3)	C32—C33—H33	119.9
C13—C12—H12	119.7	C19—C34—H34A	109.5
C11—C12—H12	119.7	C19—C34—H34B	109.5
C12—C13—C14	119.9 (4)	H34A—C34—H34B	109.5
C12—C13—Cl1	120.5 (3)	C19—C34—H34C	109.5
C14—C13—Cl1	119.5 (3)	H34A—C34—H34C	109.5
C13—C14—C15	119.3 (3)	H34B—C34—H34C	109.5
C13—C14—H14	120.4	C31—C35—H35A	109.5
C15—C14—H14	120.4	C31—C35—H35B	109.5
C14—C15—C10	121.7 (3)	H35A—C35—H35B	109.5
C14—C15—H15	119.2	C31—C35—H35C	109.5
C10—C15—H15	119.2	H35A—C35—H35C	109.5
N1—C16—C21	121.6 (3)	H35B—C35—H35C	109.5
			
C16—N1—C2—C3	149.8 (3)	C2—C10—C15—C14	176.6 (3)
C6—N1—C2—C3	−32.9 (4)	C6—N1—C16—C21	12.6 (4)
C16—N1—C2—C10	−82.0 (3)	C2—N1—C16—C21	−170.0 (3)
C6—N1—C2—C10	95.3 (3)	C6—N1—C16—C17	−166.2 (3)
N1—C2—C3—C4	44.2 (4)	C2—N1—C16—C17	11.2 (4)
C10—C2—C3—C4	−84.6 (3)	N1—C16—C17—C18	178.8 (3)
N1—C2—C3—C7	−133.7 (3)	C21—C16—C17—C18	0.0 (5)
C10—C2—C3—C7	97.6 (4)	C16—C17—C18—C19	−0.5 (5)
C28—N2—C4—C3	164.0 (3)	C17—C18—C19—C20	0.6 (5)
C28—N2—C4—C5	−14.7 (5)	C17—C18—C19—C34	−177.6 (3)
C7—C3—C4—N2	−3.8 (5)	C18—C19—C20—C21	−0.2 (5)
C2—C3—C4—N2	178.3 (3)	C34—C19—C20—C21	178.0 (3)
C7—C3—C4—C5	174.9 (3)	C19—C20—C21—C16	−0.3 (5)
C2—C3—C4—C5	−3.0 (4)	N1—C16—C21—C20	−178.4 (3)
N2—C4—C5—C6	131.0 (3)	C17—C16—C21—C20	0.4 (4)
C3—C4—C5—C6	−47.9 (4)	N1—C6—C22—C23	−28.6 (4)
C16—N1—C6—C22	−73.9 (3)	C5—C6—C22—C23	95.9 (3)
C2—N1—C6—C22	108.7 (3)	N1—C6—C22—C27	154.7 (3)
C16—N1—C6—C5	161.4 (2)	C5—C6—C22—C27	−80.8 (3)
C2—N1—C6—C5	−16.0 (4)	C27—C22—C23—C24	1.2 (5)
C4—C5—C6—N1	56.5 (3)	C6—C22—C23—C24	−175.6 (3)
C4—C5—C6—C22	−70.6 (3)	C22—C23—C24—C25	−0.7 (5)
C8—O2—C7—O1	3.0 (5)	C23—C24—C25—C26	−1.0 (5)
C8—O2—C7—C3	−176.3 (3)	C23—C24—C25—Cl2	179.4 (2)
C4—C3—C7—O1	5.2 (5)	C24—C25—C26—C27	2.2 (5)
C2—C3—C7—O1	−177.0 (3)	Cl2—C25—C26—C27	−178.3 (2)
C4—C3—C7—O2	−175.6 (3)	C25—C26—C27—C22	−1.7 (5)
C2—C3—C7—O2	2.2 (4)	C23—C22—C27—C26	0.0 (4)
C7—O2—C8—C9	174.9 (3)	C6—C22—C27—C26	176.9 (3)
N1—C2—C10—C11	−172.4 (3)	C4—N2—C28—C29	100.0 (4)
C3—C2—C10—C11	−44.8 (4)	C4—N2—C28—C33	−79.7 (5)
N1—C2—C10—C15	10.7 (4)	C33—C28—C29—C30	−1.2 (6)
C3—C2—C10—C15	138.3 (3)	N2—C28—C29—C30	179.1 (3)
C15—C10—C11—C12	−0.1 (5)	C28—C29—C30—C31	0.7 (6)
C2—C10—C11—C12	−177.2 (3)	C29—C30—C31—C32	−0.6 (7)
C10—C11—C12—C13	0.4 (5)	C29—C30—C31—C35	−179.8 (4)
C11—C12—C13—C14	−0.2 (6)	C30—C31—C32—C33	0.8 (6)
C11—C12—C13—Cl1	178.9 (3)	C35—C31—C32—C33	−179.9 (4)
C12—C13—C14—C15	−0.3 (5)	C29—C28—C33—C32	1.4 (6)
Cl1—C13—C14—C15	−179.4 (3)	N2—C28—C33—C32	−178.8 (3)
C13—C14—C15—C10	0.6 (5)	C31—C32—C33—C28	−1.3 (6)
C11—C10—C15—C14	−0.4 (5)		

### Hydrogen-bond geometry (Å, º)

**Table:**

*D*—H···*A*	*D*—H	H···*A*	*D*···*A*	*D*—H···*A*
N2—H2···O1	0.86	2.10	2.707 (4)	127
C9—H9*A*···Cl2^i^	0.96	2.74	3.698 (4)	173

Symmetry code: (i) *x*, *y*+1, *z*.
